# Perspectives on Health-Related Social Needs Screening in Primary Care Among Black and Latine Patients

**DOI:** 10.1001/jamanetworkopen.2025.29538

**Published:** 2025-08-28

**Authors:** Kristin A. Long, Alix A. Paredes Molina, Ariel O. Blakey, Dara R. Oliveira, Katherine Barahona Paz, Camila M. Mateo, Illari Cazorla-Garcia, Mathena A. Abramson, Mari-Lynn Drainoni, M. Diane McKee, Rebecca K. Rudel, Michelle K. Trivedi, Arvin Garg

**Affiliations:** 1 Department of Psychological and Brain Sciences, Boston University, Boston, Massachusetts; 2 Department of Psychology and Behavioral Health, Children’s National Hospital, Washington, DC; 3 Department of Pediatrics, University of Massachusetts Chan Medical School, UMass Memorial Children’s Medical Center, Worcester; 4 Division of General Pediatrics, Department of Pediatrics, Boston Children’s Hospital, Boston, Massachusetts; 5 Department of Pediatrics, University of California San Francisco School of Medicine, Zuckerburg San Francisco General Hospital, San Francisco; 6 Section of Infectious Diseases, Department of Medicine, Boston Medical Center & Boston University Chobanian & Avedisian School of Medicine, Boston, Massachusetts; 7 Department of Health Law Policy & Management, Boston University School of Public Health, Boston, Massachusetts; 8 Evans Center for Implementation and Improvement Sciences, Department of Medicine, Boston University Chobanian & Avedisian School of Medicine, Boston, Massachusetts; 9 Department of Family Medicine and Community Health, University of Massachusetts Chan Medical School, UMass Memorial Health, Worcester,; 10 Division of Pulmonology, Department of Pediatrics, University of Massachusetts Chan Medical School, UMass Memorial Children’s Medical Center, Worcester; 11 Child Health Equity Center, UMass Memorial Children’s Medical Center, Worcester, Massachusetts

## Abstract

**Importance:**

Screening for health-related social needs (HRSNs) has become the standard of care, but little is known about patients’ perceptions of whether and how screening and referral should be conducted, particularly among Black and Latine populations, who have variable rates of screening and resource receipt. Limited consideration of racism within HRSN frameworks and research underlies concerns that HRSN screening and referral systems may paradoxically exacerbate inequities.

**Objective:**

To characterize Black and Latine patients’ perspectives regarding (1) the suitability of HRSN screening and referral systems within primary care, (2) decision-making regarding HRSN disclosure, (3) contextual barriers and facilitators of equitable screening and referral, including processes underlying inequitable outcomes, and (4) implementation recommendations.

**Design, Setting, and Participants:**

In this qualitative study, data collected from April to August 2023 were audio-recorded, transcribed, systematically coded, and analyzed using applied thematic analysis. The study was conducted at 4 urban family medicine clinics in Worcester, Massachusetts. Participants self-identified as Black or African American and/or Latine and spoke English, Spanish, and/or Portuguese. Enrollment ceased upon reaching thematic saturation.

**Main Outcomes and Measures:**

Grounded in the Health Equity Implementation Framework, semistructured interviews probed implementation barriers and facilitators to HRSN screening and referrals. Qualitative data were stratified by race and ethnicity and language.

**Results:**

The study included 32 participants (mean [SD] age, 43 [23] years; 23 women [72%]; 9 [28%] Black; 22 [69%] Latine; 1 [3%] both), who emphasized the importance of HRSN screening and referral systems. However, mistrust in health care systems fueled reservations about disclosing HRSNs. Participants described needing to actively weigh high perceived risks (eg, loss of privacy, judgment, or government agency involvement) with low expectations of receiving resources, ultimately limiting disclosure. Within clinics, participants explained that achieving equitable screening and referrals would require transparent, bidirectional patient-practitioner communication, systematic HRSN screening protocols and accountability, and mitigation of systemic barriers that disproportionately affect minoritized patients (eg, staff shortages, time constraints, and biases).

**Conclusions and Relevance:**

In this qualitative study, Black and Latine patients described their perceptions of HRSN screening and referral systems, emphasizing compounding barriers across screening, referral, and resource receipt and underscoring how fears of negative consequences may limit disclosure of HRSNs. Together, these findings highlight how interpersonal and structural racism influence the likelihood of Black and Latine patients’ needs being recognized or addressed. An intentional approach is necessary to enhance patient trust and to implement systematic, universal, patient-centered social care systems.

## Key Points

**Question:**

How do Black and Latine patients perceive screening for health-related social needs (HRSNs) and referral systems in primary care?

**Findings:**

In this qualitative study, 32 patients described HRSN screening and referral systems as promising but emphasized barriers that disproportionately affect minoritized patients, including lack of systematic protocols, insufficient resources, and limited trust in professionals and institutions. Patients described actively weighing perceived risks with low likelihood of benefits, ultimately limiting their disclosure of HRSNs.

**Meaning:**

Compounding barriers may reduce the likelihood of Black and Latine patients’ HRSNs being recognized and addressed, suggesting the need for strategies to promote equitable implementation of social care systems.

## Teaser

This qualitative study examines the perspectives of Black and Latine patients regarding screening for health-related social needs and referral systems in primary health care.

## Introduction

Regulations from the Centers for Medicare & Medicaid Services,^1^ National Committee for Quality Assurance,^2^ and The Joint Commission^3^ have led to health-related social needs (HRSNs) screening becoming a standard of care. Despite this mandate, patients’ perceptions of HRSN screening and referral systems remain understudied.^4^ Limited available data suggest that patients generally support the idea of health care–based HRSN screening,^4,5,6^ but some worry that screening may exacerbate stigma and practitioner bias.^7,8^ Little is known about racially and ethnically minoritized patients’ perspectives on HRSN screening and referrals.^4^ This gap is critical since racially and ethnically minoritized individuals experience a disproportionate burden of HRSNs stemming from structural racism and societal factors,^9,10,11,12,13^ including economic hardship, unsafe living conditions, and reduced access to transportation and education.^10,14,15,16,17^

HRSN screening and referral systems show promise for improving social and health outcomes^18^ via resource connection, improved and tailored care plans, and feeling supported by their care team.^19^ However, rates of screening, referral, and resource connection are highly variable both within and across primary care practices.^20,21^ Lower screening rates have been documented among Latine patients^22,23,24^ and those with primary languages other than English.^24,25^ Lower screening rates also have been documented for Black patients, although findings are mixed.^22,23,24,26,27^ Practitioner-reported screening barriers include limited staff time, training, and knowledge of available resources, along with insufficient community resources.^6,20,23,28^ Families within pediatric settings describe double loss after screening—that is, disclosing sensitive HRSN information (first loss) without addressing identified needs (second loss)—thus increasing the hesitancy to engage with future screening or seek HRSN-related assistance.^29^ This is especially relevant for minoritized communities, for which pervasively inequitable care^10,11,12,15^ underlies mistrust in health care systems.^30^

Despite being a key driver of HRSNs and health,^12,31^ racism has been largely absent from HRSN frameworks and research.^10^ This absence raises concerns that without intentionally acknowledging and addressing racism, implemented HRSN screening and referral systems may paradoxically exacerbate health inequities.^4,20,32^ Furthermore, minoritized patients’ perceptions of the relevance and barriers to HRSN screening and referral remain largely unknown.^6^ This qualitative study aims to characterize Black and Latine patients’ perspectives regarding (1) the suitability of family medicine clinics for HRSN screening and referral systems, (2) how patients decide to disclose HRSNs, (3) contextual barriers and facilitators, including processes underlying inequitable outcomes, and (4) implementation recommendations.

## Methods

Findings from this qualitative study are part of a larger HRSN screening and referral effectiveness-implementation trial in urban family medicine clinics, which often serve higher numbers of minoritized and lower-income patients.^33,34^ Qualitative methods were used to understand processes (ie, how barriers affect screening and referrals) amidst interacting patient characteristics (race, ethnicity, language, and immigration).^35^ The presentation of findings adheres to the Standards for Reporting Qualitative Research (SRQR) reporting guideline.^36^ The study was approved by the UMass Chan Medical School institutional review board.

### Participants and Recruitment

Participants came from 4 family medicine clinics in Worcester, Massachusetts, each serving 7000 to 35 000 patients. Two are designated federally qualified health centers (18 participants), and 2 are located within an academic health care center (14 participants). Across sites, patients are 7% to 33% Black, 16% to 53% Latine, and 22% to 72% Medicaid recipients or uninsured. Spanish, English, and Portuguese are the top-spoken languages. Two sites were implementing social determinant of health screening using the Community Care Cooperative screener; 2 sites were piloting screening using an adapted version of the Oregon Primary Care Association screener. Across sites, resource sheets were provided following positive screens.

Adult patients and parents of pediatric patients were eligible if they were aged 18 years or older; identified as Black or African American and/or Latine or Hispanic; and spoke Spanish, Portuguese, or English. There were no exclusion criteria. Bilingual, bicultural research staff recruited participants in clinic waiting rooms. Purposive sampling ensured variability across race, ethnicity, language, and clinic affiliation; participant characteristics were periodically reviewed, and subsequent recruitment prioritized patients with characteristics underrepresented in the sample. Recruitment concluded upon reaching thematic saturation (ie, when no further themes emerged from the data).^37^

### Quantitative Measures

Participants completed a sociodemographic background questionnaire and social risk items from the Children’s HealthWatch survey^38^ (eTable 1 in Supplement 1). Four items measured energy insecurity (eg, utility shut-off).^38^ The 2-item Hunger Vital Sign^39^ captured food insecurity. Three items from the Housing Stability Vital Sign^40^ measured risk for displacement and homelessness. Positive endorsement of any item denotes risk.

### Qualitative Interviews

A semistructured interview guide (eTable 2 in Supplement 1) was informed by the Health Equity Implementation Framework, which outlines determinants of inequitable implementation.^41,42^ Interviews assessed personal experience with screening and referrals and probed decision-making processes regarding HRSN disclosure. Then, participants were asked about implementation barriers and facilitators related to screening and referral protocols, materials, and context (eg, aspects of the patient, provider, clinic, and service systems), including how and why these determinants may impact screening and referral experiences. During data collection, framework matrices were used to monitor preliminary findings, inform thematic saturation, and highlight unanswered questions. Interview guides were iteratively revised to deemphasize saturated themes and emphasize questions about unsaturated themes.

### Procedure

Data were collected between April and August 2023 in English (16 participants), Spanish (13 participants), or Portuguese (3 participants). After obtaining written or verbal informed consent, interviews were conducted by a doctoral trainee (A.O.B.) and bilingual research assistants (A.A.P.M., D.R.O., and K.B.P.), all of whom identify as racially or ethnically minoritized. Participants chose to be interviewed by telephone (16 participants) or video call (Zoom; 16 participants). Interviews (median, 69 minutes; range, 21-100 minutes) were audio-recorded, transcribed verbatim, deidentified, and checked for accuracy. An external transcription company translated Spanish and Portuguese interviews into English to facilitate coding. Afterward, quantitative surveys were completed using REDCap.^43^ Participants were compensated with $100 gift cards.

### Statistical Analysis

A coding structure (eTable 3 in Supplement 1) was developed deductively from the Health Equity Implementation Framework^41,42^ and was refined inductively to incorporate interim findings. Once the coding structure was finalized, 7 transcripts were double-coded to ensure consistency among coders. The team met weekly to resolve discrepancies and ensure fidelity to the coding structure. Transcripts were coded in NVivo 12.^44^ Data were analyzed in 2 ways. First, findings were deductively organized into the Health Equity Implementation Framework domains. Second, themes were inductively derived using applied thematic analysis,^45^ which includes preliminary familiarization with the data, systematic coding (ie, giving concise labels to portions of text), and developing, refining, and defining semantic and latent themes. Qualitative data were stratified by race, ethnicity, and language. Descriptive statistics were calculated using Excel software (Microsoft). See eMethods in Supplement 1 for positionality and reflexivity information.

## Results

The study included 32 participants (mean [SD] age, 43 [23] years; 23 women [72%]; 9 [28%] Black; 22 [69%] Latine; 1 [3%] both) (Table 1). Twenty-nine participants (91%) endorsed 1 or more HRSNs across food (26 participants [81%]), energy (17 participants [53%]), and/or housing (14 participants [44%]) insecurity (eTable 2 in Supplement 1). Specific barriers that inordinately affect minoritized patients were mapped onto the Health Equity Implementation Framework^41,42^ (Table 2). Themes illustrating the processes through which barriers lead to inequitable screening and referral (Figure) are described below and in Table 3 (summary and example quotations). Potential solutions appear in Table 4. Except where noted, findings were similar across participant race, ethnicity, language, and clinic affiliation.

**Table 1.** Participant Demographic Characteristics

Characteristic	Participants, No. (%) (N = 32)
Age, mean (SD), y	43 (12)
Gender	
Woman	23 (72)
Man	9 (28)
Birth country	
US, including Puerto Rico	14 (44)
Outside of US^a^	18 (56)
Race and ethnicity	
Black or African American and Latine or Hispanic	1 (3)
Black or African American and non-Latine or non-Hispanic	9 (28)
Latine or Hispanic and other race^b^	3 (9)
Latine or Hispanic (no specified race)	7 (22)
White and Latine or Hispanic	12 (38)
Marital status^c^	
Never married	10 (31)
Married	12 (38)
In a marriage-like relationship	7 (22)
Divorced	5 (16)
Not listed or unknown	1 (3)
Household income in past year, $^d^	
No household income in the past year	7 (22)
1-9999	6 (19)
10 000-19 999	2 (6)
20 000-29 999	5 (16)
30 000-39 999	4 (13)
40 000-49 999	3 (9)
80 000-89 999	1 (3)
Unknown	4 (13)
Highest level of completed education	
8th Grade or less	2 (6)
9th to 12th Grade; no diploma	2 (6)
High school graduate or General Educational Development	8 (25)
Vocational, trade, or business school program	1 (3)
Some college, but no degree	8 (25)
Associate’s degree	4 (13)
Bachelor’s degree	6 (19)
Unknown	1 (3)
Medical insurance^c^	
Medicaid	24 (75)
Medicare	6 (19)
Private insurance	4 (13)
No insurance or out of pocket	1 (3)
Current work situation	
Unemployed, looking for work	6 (19)
Unemployed, not looking for work (eg, disabled or student)	9 (28)
Part-time work	9 (28)
Full-time work	7 (22)
Unknown	1 (3)

^a^ Other countries included Bolivia (1 participant), Brazil (3 participants), Colombia (2 participant), Dominican Republic (4 participants), Guatemala (2 participants), Ghana (2 participants), Haiti (1 participant), Honduras (1 participant), Kenya (1 participant), and Venezuela (1 participant).

^b^ Two participants identified as Latino or Hispanic and Native American, and 1 identified as Middle Eastern.

^c^ Percentages add up to more than 100% as participants were given the option to select all that apply.

^d^ Percentages add up to more than 100% due to rounding.

**Table 2.** Findings Mapped Onto the Health Equity Implementation Framework

Level	Findings
Innovation	Overall, the HRSN screening and referral process should be systematic, universal, efficient, and discreet Leaving screening and referral to chance or staff discretion can lead to bias in who gets screened or connected to resources Universal screening removes onus from patients to proactively raise concerns (especially if unaware that resources exist) Equitable screening requires follow-through and accountability within clinics Urgency of unmet basic needs requires a rapid response to positive screening results Patients have strong concerns about privacy across all parts of the screening and referral process Screener administration: Screening should be brief due to time constraints for both patients and clinic staff or practitioners Screeners should be offered in different formats (eg, text or paper) and consider accessibility regarding language and literacy Connection with resources: Resource sheets: Information should be concise and clear, with optional opportunities for further information Resource sheets should ensure accessibility for patients across literacy levels, primary language, technology access, and level of familiarity with social services Patients prefer more active involvement than what resource sheets alone provide (emphasized more by Latine participants). Therefore, practitioners and staff should: Intentionally match patients to specific resources Facilitate connections between patients and community organizations to overcome access barriers and promote trust Ensure follow-through, making sure that resources are actually received
Recipient: patient	Patients actively consider whether and how much to disclose during HRSN screening based on: Feelings of safety and respect within the clinic Presence of unmet HRSN Fears of adverse consequences (eg, fraud, Child Protective Services involvement, or deportation) Expectations that screening will lead to desired or promised outcomes (ie, actual receipt of resources) Aspects of the patient’s identity (eg, race or language) may influence whether and how screeners are reviewed and acted upon by clinic staff Obtaining resources is challenging, and patients: May not know how to navigate community-based resources (emphasized more by Latine participants) Incur additional costs (eg, transportation or missing work to go in person; emphasized more by Latine participants) Must have self-advocacy, grit, motivation, and persistence (emphasized more by Black participants)
Recipient: provider	Personal characteristics of the personnel involved in screening and referral are more important than their position within the clinic: Across the screening and referral process: respectful, nonjudgmental, genuine desire to help, discretion (confidentiality), cultural competence After a positive screen: knowledge of resources and eligibility; adequate time and skills to facilitate connection to resources Higher practitioner and staff investment leads to higher likelihood of receiving support and resources
Clinical encounter	Clinic staff appear busy and overtaxed (a key concern that cuts across innovation, clinical encounter, and context levels) Respectful interactions with clinic staff at clinic and/or community organization (past and current) increase the likelihood that patients will complete screeners and disclose HRSNs Transparency about the screening and referral rationale and process is important when presenting the screener, including who will have access to HRSN information because it provides patients with the requisite information to decide whether and how much to disclose A positive screen is an opportunity to build trust with patients and communities, depending on the interpersonal quality of the interaction (ie, respectful and nonjudgmental) and the degree of follow-through and follow-up by clinic staff
Context	Systemic issues within the health care system affect patients’ perceptions of HRSN screening: appointments feel rushed, practitioners appear overtaxed, clinics are understaffed, and patients are unable to reach clinics outside of appointment times Navigating service systems is complicated, confusing, time-consuming, lengthy, costly, and requires a working knowledge of how these systems work These barriers are amplified for racially, ethnically, and linguistically minoritized patients, with the result that they are less likely to receive resources Patients have different levels of knowledge of US-based health and social service systems based on their immigration circumstances, and there are additional barriers for noncitizens (eg, risks of disclosure, unclear or limited eligibility; emphasized more by Latine participants) The overall shortage of available resources and long waitlists are misaligned with the urgency of patients’ unmet HRSN

Abbreviation: HRSN, health-related social need.

**Figure.**
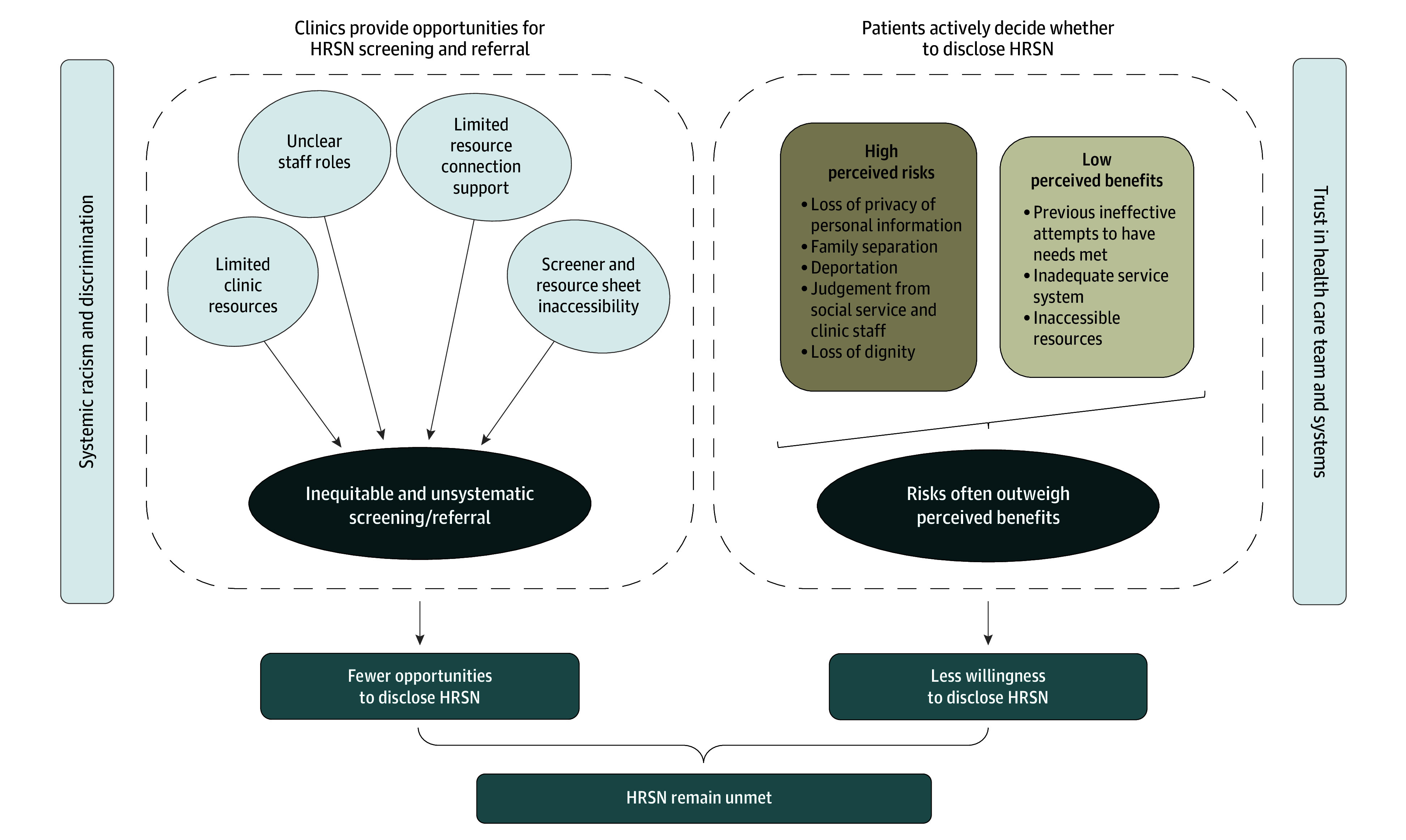
Clinic-Level and Patient-Level Influences on Disclosing and Addressing Health-Related Social Needs (HRSNs) The promise of primary care–based HRSN screening and referral is unrealized for racially and ethnically minoritized patients (theme 1). Unsystematic and inequitable screening and referral protocols within clinics limit opportunities for racially and ethnically minoritized patients to engage in HRSN screening and referral systems (theme 2). Patients actively decide whether and how much to disclose HRSNs, with high anticipated risks often outweighing limited potential benefits (theme 3). Trust in health care professionals and institutions is reduced by previous and current experiences of racism, discrimination, and unsuccessful attempts to have needs met. In turn, limited trust stemming from clinic-level and patient-level processes reduce patient disclosure of HRSNs in primary care settings, ultimately leaving HRSNs unaddressed.

**Table 3.** Example Quotations

Key points	Quotations
Theme 1: The promise of primary care-based HRSN screening and referral systems is unrealized for racially and ethnically minoritized patients	
There is high HRSN burden in the community Clinics sit at the intersection of patients and social services Patients are aware of their own needs but not of existing resources Patients believe that clinic staff know about community-based resources and services	“If I had that information [about HRSN resources], I wouldn’t be in the situation I am in right now…they woulda helped me with getting this whole housing process started. I didn’t know none of this…I had to struggle…I didn’t know that I qualify for food stamps…they would have been able to help me with all that if I would have filled out that [HRSN screener] in the beginning.” Latine, English-speaking participant “If you are a patient, you don’t know where to go…. A lot of people live paycheck to paycheck. Sometimes, even meeting your, your meal is hard, and you don’t want to go beg for food. And, if you go there [to the clinic] they will tell you, you know, you can go to this place, and they give free food…. There are people who need that food, and maybe they don’t know where to go. There are people who need shelters, they don’t know where to go…. If [clinics] don’t ask you [about HRSN], they will not know whether you have the problem. They need to ask, and they need to be clear about what could come from this kind of screener.” Black, English-speaking participant “[Clinics] could be a bridge for this person to be able to solve so many things. [Immigrants] don’t have access like the Americans…. [Staff] don’t say ‘I speak Spanish.’ They let them get frustrated because [patients] don’t know what to do there. And then, they are like, ‘Wait over there.’ ‘You didn’t do this.’ ‘Bring this paper.’ And they don’t tell you bring this, this, and that. Instead, you have to go over [to the social service organization] three times, when you could have just gone once…. You don’t know anything as an outsider.” Latine, Spanish-speaking participant^a^
Theme 2: Achieving equitable HRSN screening and referral requires systematic clinic protocols that balance efficiency with targeted patient support	
Subtheme 2.1: Achieving universal screening and resource connection requires systematic protocols and accountability	
Unclear who needs resources; thus, screening should be universal Universal screening takes the onus off of patients to bring up HRSNs Clinic-level barriers (eg, staff shortages, time constraints, and unclear roles) disproportionately affect patients with the highest needs Systematic protocols should ensure follow-through and accountability	“You never know what somebody is in need of…people silently suffer. They don’t want to ask for help. They just deal with their daily struggles…when help could be just a step away.” Black, English-speaking participant “I don’t think it [screener distribution] should be biased. I think it should be handed out to every family, because from the outside looking in, you really don’t know what someone’s, um, you know, situation is. So, I think it should be an all-around thing…. You don’t want to be stereotypical or biased against anyone. So, um, keeping it at a level where you’re sending it to every family, I think is your safest bet.” Black, English-speaking participant “[Some patients] can’t express themselves, they don’t know how to request or apply for it [HRSN support], they don’t know how to read, they don’t know how to speak English…most of your staff, I’d say 70%-90%, speak English and don’t speak other languages, so they don’t have the ease to interpret the needs of others. So, it [screening] becomes a selective process, even though it has not aimed to be selective.” Latine, Spanish-speaking participant^a^ “I think that [lack of follow-through] is just human error…. Maybe they had a family emergency or something like that, or they’re on [maternity] leave…but you [can’t] just put [HRSN] on the back burner. These people [patients], if they’re saying yes to these [HRSN] questions, they probably need immediate help…you can’t wait like weeks to help someone out with that. That’s something that you need to sort of hop on and be like, ‘Ok, here’s a food pantry, here’s the church that gives that food this such and such date….’ It’s probably gonna be important to know the staff that’s gonna be handling that, that they are going to give their attention to that. And if for some reason something does happen to that person [staff member], there’s a contingency plan.” Latine, English-speaking participant
Subtheme 2.2: Screening and referral protocols must balance efficiency with variable patient support needs and provide more active support when warranted	
Screening must be efficient, because (1) clinics and patients are busy and (2) unmet basic needs are pressing Navigating community-based service systems feels complicated, confusing, time-consuming, costly, and requires a working knowledge of how systems work Resource sheets can be overwhelming and make assumptions about literacy, language, and access to technology and transportation Some patients require more active support to access resources	“A lot of doctors are like, you know, rush, rush to get out of the appointment to go and see the next patient…they’re not trying to give you the resources that they have, because they have another patient.” Latine, English-speaking participant “What I’ve noticed is if there’s any assistance from the doctor’s office, you fill it [the paperwork] out and then give it to them, for them to submit to the, wherever it has to go, the response is very quick. But individually when you do it, and then mail it to them or even go and drop it off yourself, the effect, it doesn’t work that way…no matter what you submit, it’s not enough, you know? [Differential treatment from social services is] 100% because I am Black…. Your applications can be on somebody’s desk for weeks and it’s been, you know, maybe they haven’t even touched it yet. But if anything pops up on the screen from the doctor’s office, they have to look into it and then respond right away.” Black, English-speaking participant “Giving me a list [resource sheet] and telling me, ‘Look, you can call all these people’…. You’re not even thinking if this person can communicate in the [same] language or not. You’re not thinking about the level of literacy that the person might have…it’s not enough to just give out a list [resource sheet]…. Talk to [patients] directly, saying, ‘Okay, I want to get a good understanding of what your needs are and what kind of organization would be a good fit for you….’ [It’s] not just connecting them, but match[ing] them with organizations or programs…where you can say, ‘These companies are English only [speaking], English and Spanish [speaking]…. These [organizations] deal with the Latino community, so they’re going to be able to speak Spanish and they’re going to have that cultural competency….’ [Eventually], the [patient] population would be educated…not just knowing about [these organizations], but knowing how to navigate these structures.” Latine, Spanish-speaking participant^a^ “Some people [patients] can just take the paper [resource sheet] and do it themselves. Others need to be walked through the process. Everybody’s different.” Black, English-speaking participant
Theme 3: Patients actively weigh potential risks and benefits of disclosing HRSN during screening	
Subtheme 3.1: Strong fears about potentially life-altering negative consequences increase hesitancy to share HRSNs	
Patients have strong concerns about privacy breaches, government involvement (eg, CPS or ICE), fraud, and judgment These potentially life-altering concerns deter patients from disclosing HRSNs during screening	“If we lived in a different world, then I’d shout it from the rooftops. But, we don’t. We live in a very judgmental society…if I could easily say…‘I’m struggling’ and my community said…‘Let’s help you,’ I would tell everyone when I’m struggling. But, that’s not what they do.” Black, English-speaking participant “You hear it [staff/providers talking about patients] when you’re in the hallways. We were in the ER just this weekend [and] you could just hear the nurses talking about some other patient. Granted, they’re not name dropping. But, I’m sure if I was around long enough, I could have figured out who you were talking about. I could have figured out who was stressing you out. I could have figured out who was causing you all these inconveniences…. It makes me feel like when I go to a clinic, a hospital or a health care setting, I am trying to be the least amount of burden I can be.” Black, English-speaking participant “When I first came here [to the United States], I was so afraid of talking. And, I’m not illegal or anything, but it scared me. When you come from your country, for example, the first thing someone tells you in your country [of origin], ‘You have to [be careful], because if you’re going with kids and if you say things that…[you shouldn’t say]…they take away your kids.’” Latine, Spanish-speaking participant^a^ “All this [HRSN-related] information…should be separate and confidential…. There’s been cases where their information passes to other entities. Or when these entities require information [from clinics], well, you hear that in a lot of cases, [governmental entities] look for where [people] get medical support or food support. I think that’s why people are sometimes afraid to give their information out.” Latine, Spanish-speaking participant^a^
Subtheme 3.2: Doubts about receiving resources limit HRSN disclosure	
Previous unsuccessful attempts to access resources fuel skepticism that screening will lead to actual support Experienced and observed differential treatment toward minoritized people limits willingness to disclose HRSNs	“They make you fill out many things [that are supposed to help you] and, at the end of the day, nothing happens…promise after promise. You don’t live on promises.” Latine, Spanish-speaking participant^a^ “They[‘re] kind of gonna be like, ‘Ok, we’ll reach back to you’ and that will be it. So you keep waiting and waiting and no response, you have to call again and again…. I’m on the list [for Section 8 housing]. It’s almost [been] about eight years now, and I haven’t heard anything from them.” Black, English-speaking participant “Now, people are afraid…they think they will always be taken for fools, because that is how Hispanics think…. Like, [if] the hospital gives you [a screener] to do anything, and they don’t [follow through], people don’t fill them out. They don’t fill them out, because people are tired of the lies and fake promises that they will receive help, and they don’t help at all. You know what I mean?” Latine, Spanish-speaking participant^a^
Subtheme 3.3: Interpersonal interactions with health care staff shape decision-making regarding HRSN disclosure	
Staff interactions and clinic reputation influence whether patients feel safe sharing HRSN Positive relationships with clinic staff attenuate concerns regarding HRSN disclosure HRSN screening may promote staff-patient communication and resource connection	“Relationships [with staff] are definitely important. I think they should continue to be those same nonjudgmental, nonconfrontational types of supports…. I don’t wanna feel bad for wanting to get the help that I need…shamed about getting the help that I need…[or] any negative context coming for me trying to better myself, my life or my situation.” Black, English-speaking participant “[What affects HRSN screening participation is] having a good rapport…interact with the patient on that one-on-one level, showing your patient you care, not rushing that patient, like just giving them your time, helping them, giving them a way to contact you if they do need extra help…. Nobody’s gonna wanna go someplace and fill out a paper and people are rude to you, because that’s not gonna make you want to do nothing. That’s like, ‘Ok, they’re not trying to help me. They’re just giving me some papers.’… You’re just gonna wanna be in and out, that’s it. Do what you came there to do and go. You’re not gonna be wanting to ask for help.” Latine, English-speaking participant

Abbreviations: CPS, Child Protective Services; ER, emergency room; HRSN, health-related social need; ICE, Immigration and Customs Enforcement.

^a^ These quotations were originally in Spanish and translated into English by bilingual and bicultural research staff.

**Table 4.** Participant-Identified Strategies for Effective Implementation of HRSN Screening

Recommendation^a^	Examples
Provide transparency regarding screening process	Clearly explain the rationale of HRSN screening: identifying patient social needs and connecting them with relevant community resources Educate both patients and clinic staff on the entire HRSN screening process, including what questions are being asked, how responses are reviewed, availability of resource referrals, and expected timelines of processing Inform patients on how their responses are stored, who will specifically have access to their information (eg, designated clinic staff, community resource centers, or government agencies), and how this access is managed both within and outside the clinics
Promote patient autonomy	Present screener as optional, emphasizing patients can share only as much as they are comfortable disclosing Only ask for necessary information on HRSN screeners, and design or select screeners which allow patients to share more details if they choose Provide HRSN resource information (eg, pamphlets) to all patients, regardless of their participation in the screening
Prioritize patient privacy	Ensure patients have a private area within the clinic to ask about HRSN screening, resources, and complete screening Offer opportunities to privately discuss patients’ hesitations to participating in HRSN screening and provide honest information regarding their concerns, such as: Unwanted governmental authority involvement (eg, Immigration and Customs Enforcement or Child Protective Services) Judgment from staff, other patients, or resource organizations Unauthorized disclosure of personal information (eg, address or Social Security number) Establish protocols that actively safeguard HRSN data and are regularly evaluated for effectiveness, ensuring sensitivity and discretion are prioritized when handling screening responses
Promote respectful interpersonal relationships	Center empathy and respect in communication with patients before, during, and after screening through: Recognizing and addressing potential stigmas related to social risk needs, acknowledging its hurtful impact Active listening, fully hearing and acknowledging patient concerns without interrupting Nonjudgmental attitudes, responding to patients’ disclosures directly and with understanding, rather than blame or criticism Normalizing the presence of hard times and emphasizing potential solutions Following up with patients regarding the status of their screener, emphasizing that their responses matter and demonstrating the clinic’s commitment to their care
Tailor screening and referral process to maximize accessibility	Ensure accessibility of HRSN screeners by using simple language (ie, avoid jargon), incorporating visual aids, not requiring access to computers or internet, and developing protocols to support patients with low literacy Provide screening and related resource information in multiple languages Consider patients’ race, ethnicity, cultural background, and spoken language when selecting and administering HRSN screeners Recruit staff who reflect the clinic’s patient demographic to better support diverse patient needs Ensure support services on resource sheets are culturally and linguistically relevant (eg, informing patients of languages spoken at resource sites) Offer clear guidance on accessing transportation, interpretation, and internet support for community resources on resource sheets

Abbreviation: HRSN, health-related social need.

^a^ The goal is to improve HRSN screening accessibility and enhance patient trust to promote informed HRSN disclosure and appropriate referrals for community-based resources.

### Theme 1: The Promise of Primary Care–Based HRSN Screening and Referral Systems Is Unrealized for Racially and Ethnically Minoritized Patients

Participants supported health care–based screening and referral systems, emphasizing how HRSNs frequently and negatively affect health and well-being, particularly among racially and ethnically minoritized communities. Yet, they described limited familiarity with screening and little awareness of what resources exist, how to identify or access resources, or whether clinics could facilitate resource access. Primary care was identified as uniquely positioned to connect patients with community-based resources given beliefs that clinic personnel know about community-based resources and patients’ needs. However, participants described how clinic protocols, interpersonal interactions, and perceived resource inaccessibility may limit racially and ethnically minoritized patients’ opportunities and willingness to disclose and/or address HRSNs—emphasizing the foundational importance of interpersonal and institutional trust.

### Theme 2: Achieving Equitable HRSN Screening and Referral Requires Systematic Clinic Protocols That Balance Efficiency With Targeted Patient Support

#### Theme 2.1: Achieving Universal Screening and Resource Connection Requires Systematic Protocols and Accountability

Because HRSNs are not always apparent, participants emphasized that all patients should be screened but also articulated how clinic-level barriers may undermine universal screening. Participants observed how staff and practitioners appear rushed and overtaxed, which they attributed to staff shortages and time constraints. Similarly, they observed disorganization and unclear staff roles, both regarding HRSN protocols and more generally. Given limited staff time and resources, participants explained how time-saving shortcuts may bias who gets screened or connected with resources. For example, HRSNs may increase transportation difficulties, and arriving late decreases time for extra initiatives like HRSN screening or referral. Interpreter use substantially increases appointment duration and/or reduces content, ultimately limiting the time for screening. Few bilingual staff or inattention to language within screening and referral protocols may lead to non-English screeners being skipped, delayed, or disregarded. Even when screening is completed, participants wondered whether staff bias may lead to reduced responsiveness toward minoritized patients. These expectations stem from observations, personal experiences, and/or community experiences of unequal treatment based on race or ethnicity and nonresponsiveness to previous requests for support. Altogether, participants considered HRSN screening and referral to be a privilege unavailable to some patients.

Participants considered ways to mitigate biases in screener administration, review, and/or response and recommended predetermined, systematic processes that apply to all patients. They suggested incorporating universal screening into routine care and developing tracking systems to monitor which patients are missed, emphasizing the importance of follow-through and accountability. Participants cautioned against having 1 designated point person to ensure continuity amidst staff changes (eg, turnover or childbirth leaves). Finally, they explained how systematic protocols may remove the onus from patients to proactively disclose HRSNs, especially when unaware that resources exist. Overall, participants noted that with truly systematic protocols, screening or referral should not depend on patient characteristics, chance, or staff discretion.

#### Theme 2.2: Screening and Referral Protocols Must Balance Efficiency With Variable Patient Support Needs and Provide More Active Support When Warranted

With regard to balancing efficiency with variable support needs, participants emphasized the importance of efficiency to accommodate clinic constraints (eg, overtaxed practitioners) and address urgent HRSNs. Prioritizing attainability and sustainability, participants envisioned universal screening and referral systems fitting within clinic workflows, without shortchanging discussions of medical concerns. Patients’ own time constraints, which are often amplified by HRSNs, similarly necessitate brief screening conducted during existing visits. After screening, the urgency of HRSNs (eg, food insecurity) requires timely responses.

Yet, participants also explained how some patients require more active support. Regarding screening, they emphasized that both screeners and screening protocols should account for differences across patient knowledge, literacy, language, and technology access. Regarding community-based resources, participants emphasized that navigating social service systems is complicated, confusing, time-consuming, and costly, requiring working knowledge of how systems work. Many participants, particularly Latine patients and/or immigrants, described not knowing where to start, including whom to contact or what to say. Roadblocks included ineligibility for services, incorrect paperwork, unavailability during work hours, and limited access to internet, telephone, transportation, or bilingual staff and/or materials. Participants strongly doubted that clinics would help troubleshoot these roadblocks. They explained that high motivation and sustained effort are often insufficient to overcome multiple barriers to obtaining resources; feeling lost and lacking direction about how to move forward, patients eventually stop trying.

Given these substantial barriers, participants explained that standard resource sheets may not always be sufficient. They described a tension between the potential utility vs overwhelming nature of comprehensive resource sheets, which assume literacy, technology access, transportation, and knowledge of how community-based systems operate. Although resource sheets were available in numerous languages, participants noted that community-based staff and resources are often English only. Thus, participants explained that some patients, particularly those with higher HRSNs, may need support beyond resource sheets.

Despite being more time and resource intensive, participants emphasized the importance of more active support for patients with lower English proficiency, literacy, or familiarity with US-based systems; disabilities; limited telephone or internet or transportation; or unstable housing. Ideally, participants envisioned dedicated resource support teams within clinics. Alternately, participants described a stepped approach based on patients’ abilities to independently access community-based resources: (1) provide resource sheets after positive screens; (2) orient patients to relevant information on resource sheets, highlighting specific resources matching their circumstances and outlining next steps; (3) facilitate connections between patients and community organizations to alleviate patients’ concerns about how to frame their needs and ask relevant questions, reduce the likelihood of being dismissed (clinic staff are perceived to have greater respect within communities), assuage fears of disclosing personal information to unfamiliar organizations, and promptly identify and problem-solve access barriers; and (4) follow-up regarding resource receipt and problem-solve as needed. Follow-up and personalized support were especially emphasized by Latine participants.

Participants repeatedly and emphatically described how active support could facilitate timely and equitable resource connection and, in turn, address HRSNs. Furthermore, participants, particularly immigrants, explained how the screening and referral process could promote independence by educating patients about how to identify and access available resources.

### Theme 3: Patients Actively Weigh Potential Risks and Benefits of Disclosing HRSNs During Screening

Enthusiasm for screening was tempered by concerns about potentially life-altering consequences and unlikely benefit, even in situations of high HRSNs. After actively weighing potential costs and benefits, participants often concluded that HRSN disclosure was not worth the risks, explaining how racially and ethnically minoritized patients regularly encounter bias and discrimination within systems of care.

#### Theme 3.1: Strong Fears About Potentially Life-Altering Negative Consequences Increase Hesitancy to Share HRSNs

Participants described strong fears regarding potential negative consequences of HRSN disclosure, including breached privacy and security, unwanted involvement of government agencies, judgment, threats to personal dignity, and internalized failure and shame related to disclosed needs. Participants emphasized how intense, pervasive anxieties ultimately inhibit HRSN disclosure during screening.

Privacy and security concerns were most prominent, given strong fears about who can access personal information. The seriousness of potential consequences amplified these fears. For example, participants feared that disclosing HRSNs would signal the inability to care for their children, prompting involvement from Child Protective Services and ultimately causing separation of children from their families. Similarly, participants feared that disclosing any personal information invites fraud, scams, or identity theft. Although discussed by both immigrants and US-born participants, immigrants’ limited knowledge of US culture intensified fears of being scammed.

Latine participants also feared that disclosure of HRSNs could alert Immigration and Customs Enforcement and lead to deportation of themselves or their loved ones; this pervasive fear was voiced regardless of participants’ secure legal status. Concerns stemmed from warnings from family and community members and by nationwide examples of family separation, mass deportation, and discrimination against Latine individuals. Such explicit and implicit messaging intensified fears that information shared with clinics may be released to outside entities and used against Latine patients.

Beyond threatened privacy, Black participants, in particular, explained how HRSN disclosure would elicit judgment from health care practitioners and staff, compromising patient dignity. Participants described how the longstanding nature of patient-practitioner relationships compounds fears of being perceived as inferior for having HRSNs, thereby harming patient-practitioner relationships and negatively affecting health care quality. They further described internalizing judgment, whereby values of self-sufficiency preclude admitting need and/or accepting support, leading to feelings of personal failure and shame.

#### Theme 3.2: Doubts About Receiving Resources Limit HRSN Disclosure

Although screening aims to connect patients with resources, participants doubted they would receive them. These expectations stemmed from previous unsuccessful attempts to address HRSNs through clinics or other organizations. Although some participants discussed clinic staffs’ successful advocacy to address basic needs, most described disclosing unmet social needs and/or applying for services without benefit. Black participants particularly emphasized that working hard to meet their own needs is better than wasting time interfacing with an inefficient system with little expectation of gain.

Doubts were intensified by general impressions that social service systems are inaccessible, inequitable, and poorly equipped to meet the community’s needs. Participants highlighted the mismatch between high levels of HRSNs within their communities and insufficient resources to meet those needs, underscoring how multilevel barriers reduce resource availability and accessibility for those most in need. Latine participants expressed greater doubts about actually receiving resources given uncertainty about eligibility regarding income, citizenship, and immigration status. Altogether, participants described how strong skepticism lowers their likelihood of disclosing HRSNs.

#### Theme 3.3: Interpersonal Interactions With Health Care Staff Shape Decision-Making Regarding HRSN Disclosure

Limited interpersonal trust and respect from clinic staff magnified expectations of high risks and low benefits from HRSN disclosure. Participants described how all interactions with staff or practitioners, regardless of professional role, impact patients’ expectations regarding privacy, professionalism, and commitment to patients’ well-being. These expectations strongly influence HRSN-related decision-making. Previous and current negative interpersonal interactions fueled doubts about staff’s sincerity, integrity, discretion, and respect for their patients. For example, observing staff (mis)handling patients’ sensitive information, such as overhearing discussions of medical and/or social circumstances, intensified skepticism that sensitive information remains private. Coupled with their own or others’ experiences of Child Protective Services involvement, deportation, or fraud, the careless handling of patient information reinforced perceptions that negative consequences of sharing HRSNs could become a reality.

In contrast, positive interactions and relationships with health care staff and/or practitioners enhance trust, assuage perceived risks, and bolster expectations of resource connection, which together increases patients’ likelihood of disclosing HRSNs. Beyond individual experiences, participants described how the clinic’s reputation in the community similarly informs (mis)trust and shapes HRSN disclosure. Finally, participants explained how screening may catalyze bidirectional HRSN-related communication over time, deepening practitioners’ knowledge of patients’ social circumstances and fostering the trust and continuity necessary to effectively address patients’ needs.

## Discussion

This qualitative study describes perceptions of HRSN screening and referral systems among Black and Latine patients, populations that disproportionately experience HRSNs related to structural racism^9,10,11,12^ yet remain underrepresented in the HRSN literature.^4,20,32^ Participants supported clinic-based screening and referral systems to alleviate high community HRSN burden but emphasized how poor implementation may exacerbate race-based and ethnicity-based inequities. Black and Latine patients anticipate compounding barriers across screening, referral, and resource access—magnified for patients with primary languages other than English—which exemplify systemic racism and discrimination.^46^ The combination of limited clinic resources (bilingual staff, time, and funds) and poorly designed and/or implemented protocols may prompt shortcuts affecting who gets screened and connected with resources. Even when screened, patients describe actively weighing potential risks and benefits of HRSN disclosure, often concluding that potentially life-changing risks outweigh the low likelihood of benefits. The net result is that Black and Latine patients are more likely to have HRSNs but may be less likely to have their needs recognized or addressed, thus exacerbating inequities.^9^

These findings extend the extant literature, which predominantly reflects practitioner-reported barriers.^6,20,23,28^ Despite feeling that they are on the outside, patients in this study provided key insights about systems-level implementation barriers in low-resource settings. For example, patient concerns regarding bias echo practitioner-reported findings that screening is not universal, but rather, driven by patient characteristics (eg, appearance or insurance status),^47^ underscoring the need for protocols that actively counteract bias and discrimination. For instance, setting goals regarding the percentage of patients being screened, without also monitoring the characteristics of patients being missed, may increase overall screening rates but deepen inequities. Furthermore, screening alone, without also facilitating resource connection, is unlikely to improve HRSNs. After positive screens, referral uptake ranges from 3% to 75%, with higher uptake corresponding to more active referral protocols.^21^

These findings underscore the foundational importance of interpersonal interactions, relationships, and trust in health care teams for Black and Latine patients.^8,48,49^ We extend existing research by describing decision-making processes, documenting how privacy concerns and low trust may affect HRSN disclosure. Building on the concept of double loss (disclosing sensitive HRSN information without receiving support),^29^ a potential third loss emerges, whereby poorly executed screening may intensify doubts that health care teams are committed to supporting their needs. However, these findings also illuminate the potential for intentional, authentic patient-centered HRSN screening and referral systems to deepen patients’ relationships with staff and practitioners, which aligns with HRSN screening logic models emphasizing benefits of screening beyond direct referrals to community resources.^19^

Key barriers likely to reinforce race-based and ethnicity-based inequities (eg, staff resources and trust) may be particularly challenging to address. These findings highlight the fundamental importance of personalizing support and following through on positive screens to facilitate resource receipt and build trust with patients and communities. However, these active approaches require staff time. There have been explicit calls for more infrastructure and capacity-building investments to enable equitable screening and referral implementation, particularly in underresourced settings.^10,23^ For example, social service support from community health workers^20^ may alleviate practitioners’ concerns about their own limited knowledge of community-based resources.^6^ More broadly, social care models should address the social determinants of population health, which disproportionately affect minoritized communities and constrain the possible benefits of individual social care interventions.^46,50^

### Limitations

Strengths of this research include specifically focusing on Black and Latine patients, enrolling a sample with more than 90% reporting HRSNs, conducting both inductive and deductive qualitative analyses, and grounding in an implementation equity framework.^41,42^ Limitations include unknown generalizability outside these family medicine clinics. Few participants recalled personal involvement in screening, which aligns with lower screening rates in minoritized patients^22,23,24,26,27^ but means that some findings reflect broader health care and social service experiences rather than HRSN screening and referral specifically. Future research should engage health care staff to better understand systemic challenges, including the absence vs poor implementation of HRSN tracking mechanisms.

## Conclusions

This study is timely given recent HRSN screening mandates and concerns that poor implementation^20,21^ may exacerbate existing race-based and ethnicity-based inequities.^4,20,32^ The findings inform the development of systematic, universal, patient-centered HRSN screening and referral protocols to mitigate systemic barriers for minoritized patients, consistent with the goal of effectively bridging patients and social service systems without exacerbating existing inequities.

## Notes

Supplement 1.
eMethods.
**eTable 1.** Participant Endorsement of Health-Related Social Needs (HRSN) **eTable 2.** Qualitative Interview Questions **eTable 3.** Codebook
eReferences

Supplement 2.
Data Sharing Statement

## References

[1] Jacobs DB, Schreiber M, Seshamani M, Tsai D, Fowler E, Fleisher LA. Aligning quality measures across CMS—the universal foundation. N Engl J Med. 2023;388(9):776-779. doi:10.1056/NEJMp2215539 36724323

[2] Reynolds A. Social need: new HEDIS measure uses electronic data to look at screening, intervention. November 2, 2022. Accessed January 29, 2025. https://www.ncqa.org/blog/social-need-new-hedis-measure-uses-electronic-data-to-look-at-screening-intervention/

[3] The Joint Commission. R^3^ report: requirement, rationale, reference. June 20, 2022. Accessed January 17, 2025. https://www.jointcommission.org/-/media/tjc/documents/standards/r3-reports/r3_disparities_july2022-6-20-2022.pdf

[4] Brown EM, Loomba V, De Marchis E, Aceves B, Molina M, Gottlieb LM. Patient and patient caregiver perspectives on social screening: a review of the literature. J Am Board Fam Med. 2023;36(1):66-78. doi:10.3122/jabfm.2022.220211R1 36759136

[5] Rogers AJ, Hamity C, Sharp AL, Jackson AH, Schickedanz AB. Patients’ attitudes and perceptions regarding social needs screening and navigation: multi-site survey in a large integrated health system. J Gen Intern Med. 2020;35(5):1389-1395. doi:10.1007/s11606-019-05588-1 31898132 PMC7210366

[6] Trochez RJ, Sharma S, Stolldorf DP, . Screening health-related social needs in hospitals: a systematic review of health care professional and patient perspectives. Popul Health Manag. 2023;26(3):157-167. doi:10.1089/pop.2022.0279 37092962 PMC10278007

[7] Drake C, Batchelder H, Lian T, . Implementation of social needs screening in primary care: a qualitative study using the health equity implementation framework. BMC Health Serv Res. 2021;21(1):975. doi:10.1186/s12913-021-06991-3 34530826 PMC8445654

[8] Broaddus-Shea ET, Jimenez-Zambrano A, Holliman BD, Connelly L, Huebschmann AG, Nederveld A. Unpacking patient perspectives on social needs screening: a mixed methods study in western Colorado primary care practices. Patient Educ Couns. 2024;125:108298. doi:10.1016/j.pec.2024.108298 38735120

[9] Garg A, LeBlanc A, Raphael JL. Inadequacy of current screening measures for health-related social needs. JAMA. 2023;330(10):915-916. doi:10.1001/jama.2023.13948 37603327

[10] Vasan A, Dalembert G, Garg A. An antiracist approach to social care integration. Pediatrics. 2024;153(1):e2023062109. doi:10.1542/peds.2023-062109 38058202 PMC11212503

[11] Town M, Eke P, Zhao G, . Racial and ethnic differences in social determinants of health and health-related social needs among adults—behavioral risk factor surveillance system, United States, 2022. MMWR Morb Mortal Wkly Rep. 2024;73(9):204-208. doi:10.15585/mmwr.mm7309a3 38451870 PMC10932584

[12] Paradies Y, Ben J, Denson N, . Racism as a determinant of health: a systematic review and meta-analysis. PLoS One. 2015;10(9):e0138511. doi:10.1371/journal.pone.0138511 26398658 PMC4580597

[13] National Academies of Sciences, Engineering, and Medicine; Health and Medicine Division; Board on Population Health and Public Health Practice; Board on Health Care Services; Committee on Unequal Treatment Revisited: The Current State of Racial and Ethnic Disparities in Health Care. Nass SJ, Amankwah FK, DeVoe JE, Benjamin GC, eds. Ending Unequal Treatment: Strategies to Achieve Equitable Health Care and Optimal Health for All. National Academies Press; 2024. 38924457

[14] Hood CM, Gennuso KP, Swain GR, Catlin BB. County health rankings: relationships between determinant factors and health outcomes. Am J Prev Med. 2016;50(2):129-135. doi:10.1016/j.amepre.2015.08.024 26526164

[15] National Academies of Sciences, Engineering, and Medicine, Health and Medicine Division; Board on Population Health and Public Health Practice; Committee on Community-Based Solutions to Promote Health Equity in the United States. Communities in Action: Pathways to Health Equity. National Academies Press; 2017. 28418632

[16] Egede LE, Walker RJ, Williams JS. Addressing structural inequalities, structural racism, and social determinants of health: a vision for the future. J Gen Intern Med. 2024;39(3):487-491. doi:10.1007/s11606-023-08426-7 37740168 PMC10897090

[17] Bailey ZD, Krieger N, Agénor M, Graves J, Linos N, Bassett MT. Structural racism and health inequities in the USA: evidence and interventions. Lancet. 2017;389(10077):1453-1463. doi:10.1016/S0140-6736(17)30569-X 28402827

[18] Gottlieb LM, Wing H, Adler NE. A systematic review of interventions on patients’ social and economic needs. Am J Prev Med. 2017;53(5):719-729. doi:10.1016/j.amepre.2017.05.011 28688725

[19] Gottlieb LM, Hessler D, Wing H, Gonzalez-Rocha A, Cartier Y, Fichtenberg C. Revising the logic model behind health care’s social care investments. Milbank Q. 2024;102(2):325-335. doi:10.1111/1468-0009.12690 38273221 PMC11176407

[20] Marchis EH, Aceves BA, Brown EM, Loomba V, Molina MF, Gottlieb LM. Assessing implementation of social screening within US health care settings: a systematic scoping review. J Am Board Fam Med. 2023;36(4):626-649. doi:10.3122/jabfm.2022.220401R1 37468216

[21] Ruiz Escobar E, Pathak S, Blanchard CM. Screening and referral care delivery services and unmet health-related social needs: a systematic review. Prev Chronic Dis. 2021;18:E78. doi:10.5888/pcd18.200569 34387188 PMC8388203

[22] Torres CIH, Gold R, Kaufmann J, . Social risk screening and response equity: assessment by race, ethnicity, and language in community health centers. Am J Prev Med. 2023;65(2):286-295. doi:10.1016/j.amepre.2023.02.018 36990938 PMC10652909

[23] De Marchis EH, Aceves B, Razon N, Chang Weir R, Jester M, Gottlieb LM. “Wanting the best for our folks”—a mixed methods analysis of community health center social risk screening initiatives. J Am Board Fam Med. 2023;36(5):817-831. doi:10.3122/jabfm.2023.230099R1 37775320

[24] Cottrell EK, Dambrun K, Cowburn S, . Variation in electronic health record documentation of social determinants of health across a national network of community health centers. Am J Prev Med. 2019;57(6)(suppl 1):S65-S73. doi:10.1016/j.amepre.2019.07.014 31753281

[25] Gold R, Bunce A, Cowburn S, . Adoption of social determinants of health EHR tools by community health centers. Ann Fam Med. 2018;16(5):399-407. doi:10.1370/afm.2275 30201636 PMC6131002

[26] Savitz ST, Nyman MA, Kaduk A, Loftus C, Phelan S, Barry BA. Association of patient and system-level factors with social determinants of health screening. Med Care. 2022;60(9):700-708. doi:10.1097/MLR.0000000000001754 35866557

[27] Bleacher H, Lyon C, Mims L, Cebuhar K, Begum A. The feasibility of screening for social determinants of health: seven lessons learned. Fam Pract Manag. 2019;26(5):13-19. 31502814

[28] Rudisill AC, Eicken MGA, Gupta D, . Patient and care team perspectives on social determinants of health screening in primary care: a qualitative study. JAMA Netw Open. 2023;6(11):e2345444. doi:10.1001/jamanetworkopen.2023.45444 38015502 PMC10685887

[29] Schleifer D, Diep A, Grisham K. It’s about trust: parents’ perspectives on pediatricians screening for social needs. United Hospital Fund. June 24, 2019. Accessed January 20, 2025. https://uhfnyc.org/publications/publication/its-about-trust-SDH/

[30] Bazargan M, Cobb S, Assari S. Discrimination and medical mistrust in a racially and ethnically diverse sample of California adults. Ann Fam Med. 2021;19(1):4-15. doi:10.1370/afm.2632 33431385 PMC7800756

[31] Williams DR, Lawrence JA, Davis BA. Racism and health: evidence and needed research. Annu Rev Public Health. 2019;40(1):105-125. doi:10.1146/annurev-publhealth-040218-043750 30601726 PMC6532402

[32] Garg A, Boynton-Jarrett R, Dworkin PH. Avoiding the unintended consequences of screening for social determinants of health. JAMA. 2016;316(8):813-814. doi:10.1001/jama.2016.9282 27367226

[33] Ferrer RL. Pursuing equity: contact with primary care and specialist clinicians by demographics, insurance, and health status. Ann Fam Med. 2007;5(6):492-502. doi:10.1370/afm.746 18025486 PMC2094023

[34] Grumbach K, Hart LG, Mertz E, Coffman J, Palazzo L. Who is caring for the underserved? a comparison of primary care physicians and nonphysician clinicians in California and Washington. Ann Fam Med. 2003;1(2):97-104. doi:10.1370/afm.49 15040439 PMC1466573

[35] Hamilton AB, Finley EP. Qualitative methods in implementation research: an introduction. Psychiatry Res. 2019;280:112516. doi:10.1016/j.psychres.2019.112516 31437661 PMC7023962

[36] O’Brien BC, Harris IB, Beckman TJ, Reed DA, Cook DA. Standards for reporting qualitative research: a synthesis of recommendations. Acad Med. 2014;89(9):1245-1251. doi:10.1097/ACM.0000000000000388 24979285

[37] Saunders B, Sim J, Kingstone T, . Saturation in qualitative research: exploring its conceptualization and operationalization. Qual Quant. 2018;52(4):1893-1907. doi:10.1007/s11135-017-0574-8 29937585 PMC5993836

[38] Frank DA, Casey PH, Black MM, . Cumulative hardship and wellness of low-income, young children: multisite surveillance study. Pediatrics. 2010;125(5):e1115-e1123. doi:10.1542/peds.2009-1078 20385641

[39] Hager ER, Quigg AM, Black MM, . Development and validity of a 2-item screen to identify families at risk for food insecurity. Pediatrics. 2010;126(1):e26-e32. doi:10.1542/peds.2009-3146 20595453

[40] Sandel M, Sheward R, Ettinger de Cuba S, . Unstable housing and caregiver and child health in renter families. Pediatrics. 2018;141(2):e20172199. doi:10.1542/peds.2017-2199 29358482

[41] Woodward EN, Singh RS, Ndebele-Ngwenya P, Melgar Castillo A, Dickson KS, Kirchner JE. A more practical guide to incorporating health equity domains in implementation determinant frameworks. Implement Sci Commun. 2021;2(1):61. doi:10.1186/s43058-021-00146-5 34090524 PMC8178842

[42] Woodward EN, Matthieu MM, Uchendu US, Rogal S, Kirchner JE. The health equity implementation framework: proposal and preliminary study of hepatitis C virus treatment. Implement Sci. 2019;14(1):26. doi:10.1186/s13012-019-0861-y 30866982 PMC6417278

[43] Harris PA, Taylor R, Thielke R, Payne J, Gonzalez N, Conde JG. Research electronic data capture (REDCap)—a metadata-driven methodology and workflow process for providing translational research informatics support. J Biomed Inform. 2009;42(2):377-381. doi:10.1016/j.jbi.2008.08.010 18929686 PMC2700030

[44] QSR International Pty Ltd. NVivo qualitative data analysis software. Version 12, 2018. Accessed July 25, 2025. https://help-nv.qsrinternational.com/20/win/Content/welcome.htm

[45] Guest G, MacQueen KM, Namey EE. Applied Thematic Analysis. Sage Publications; 2011.

[46] Crear-Perry J, Correa-de-Araujo R, Lewis Johnson T, McLemore MR, Neilson E, Wallace M. Social and structural determinants of health inequities in maternal health. J Womens Health (Larchmt). 2021;30(2):230-235. doi:10.1089/jwh.2020.8882 33181043 PMC8020519

[47] Wallace AS, Luther BL, Sisler SM, Wong B, Guo JW. Integrating social determinants of health screening and referral during routine emergency department care: evaluation of reach and implementation challenges. Implement Sci Commun. 2021;2(1):114. doi:10.1186/s43058-021-00212-y 34620248 PMC8499465

[48] Byhoff E, De Marchis EH, Hessler D, . Part II: A qualitative study of social risk screening acceptability in patients and caregivers. Am J Prev Med. 2019;57(6)(suppl 1):S38-S46. doi:10.1016/j.amepre.2019.07.016 31753278 PMC6876708

[49] Schoenthaler A, Hassan I, Fiscella K. The time is now: Fostering relationship-centered discussions about patients’ social determinants of health. Patient Educ Couns. 2019;102(4):810-814. doi:10.1016/j.pec.2018.10.025 30391299

[50] Kindig D, Stoddart G. What is population health? Am J Public Health. 2003;93(3):380-383. doi:10.2105/AJPH.93.3.380 12604476 PMC1447747

