# Supplementary material for: Perspectives on Health-Related Social Needs Screening in Primary Care Among Black and Latine Patients
Source: JAMA Netw Open. 2025 Aug 28;8(8):e2529538. doi: 10.1001/jamanetworkopen.2025.29538 (PMC12395316; doi:10.1001/jamanetworkopen.2025.29538)
Supplement: Supplement 1. — eMethods. eTable 1. Participant Endorsement of Health-Related Social Needs (HRSN) eTable 2. Qualitative Interview Questions eTable 3. Codebook eReferences [file jamanetwopen-e2529538-s001.pdf]

Supplemental Online Content

Long KA, Molina AP, Blakey AO, et al. Perspectives on health-related social needs screening in primary care among Black and Latine patients. *JAMA Netw Open.* 2025;8(8):e2529538. doi:10.1001/jamanetworkopen.2025.29538

eMethods

eTable 1. Participant Endorsement of Health-Related Social Needs (HRSN)

eTable 2. Qualitative Interview Questions

eTable 3. Codebook

eReferences

This supplemental material has been provided by the authors to give readers additional information about their work.

© 2025 Long KA et al. *JAMA Network Open*.

eMethods

Additional Details Regarding Procedure:

Completion of REDCap Surveys*:* Participants were given the option of completing surveys independently in REDCap or with the involvement of a research team member. If they chose the latter, the research team member either shared their screen with survey questions on Zoom and went through each question with participants, or verbally read aloud survey questions to participants via phone calls, depending on the participant's preference. During the survey administration, the research team member addressed any questions regarding survey prompts and recorded the participant's responses via REDCap.

Review of Example Screeners and Resource Sheets During Interviews*:* Participants who completed interviews over Zoom with video access were shown examples of screeners via shared screen while interviewers provided an in-depth verbal description of the screeners. Participants who completed interviews using audio only (via phone or Zoom) were provided with the same in-depth description of the screeners – e.g., screening questions and description of the screener format. Regarding resource sheets, participants were either shown an example of a resource sheet via Zoom or were given a brief verbal description of the content and format of a resource sheet (e.g., "sheets with resources related to a patient's disclosed HRSN and where they lived, such as information about food banks in your community or close to the clinic…").

Research Team Positionality and Reflexivity

The research team included post-baccalaureate research assistants, doctoral trainees, and doctoral-level researchers and clinicians with expertise spanning clinical psychology, medicine, pediatrics, implementation science, and public health. Authors identify as Latine, Black, Asian, and White, including several first-generation, immigrant, bilingual, and/or bicultural individuals. These varied

© 2025 Long KA et al. *JAMA Network Open*.

backgrounds provided complementary insights across all stages of the current research project. Research team members did not have any prior professional relationships with participants.

To enhance reflexivity, the research team engaged in ongoing discussions to critically examine assumptions, particularly surrounding how personal identities and lived experiences shaped the study design, data analysis and interpretation, and dissemination of findings. For example, these discussions explored how researchers' backgrounds informed expectations about participants' perspectives of HRSN, decisions to disclose HRSN, level of familiarity and knowledge of clinic processes, and their experiences of racism and/or discrimination in medical or social service settings. During weekly meetings, members of the coding and analysis team discussed interview content, identified potential biases, and re-centered study goals. In bi-weekly larger project meetings, the research team collectively examined interim findings, interrogated assumptions, and considered alternative interpretations of the data. Mindful of our positionality, the research team took deliberate steps to center participants' experiences rather than impose our own narratives, including peer debriefing and engagement with existing literature to contextualize present findings.

© 2025 Long KA et al. *JAMA Network Open*.

eTable 1: Participant Endorsement of Health-Related Social Needs (HRSN)

HRSN Measures and Items [a]	Endorsement of risk item, Number (%)	Non-endorsement of risk item, Number (%)	Unknown, Number (%)	Endorsement of any risk item(s) within category Number (%)	Endorsement of any risk item(s) across categories, Number (%)
**Food Insecurity:** *The two-item Hunger Vital Sign*[1]				26 (81)	29 (91)
We worried whether our food would run out before we got money to buy more. (past 12 months)	25 (78)	5 (16)	2 (6)		
The food that we bought just didn't last and we didn't have money to get more. (past 12 months)	19 (59)	12 (38)	1 (3)		
**Energy Insecurity:** *Children's HealthWatch Survey*[2]				17 (53)	
In the past year, has the utility company threatened to shut off your service for not paying your bills?	12 (38)	19 (59)	1 (3)		
In the last 12 months, were there any days that your home was not heated because you couldn't pay the bills?	4 (13)	27 (84)	1 (3)		
Have you used your cooking stove for heat?	10 (31)	21 (66)	1 (3)		
**Housing Insecurity:** *Housing Stability Vital Sign*[3]				14 (44)	
Within the past 12 months, have you ever stayed: outside, in a car, in a tent, in an overnight shelter, or temporarily in someone else's home (i.e. couch-surfing)?	5 (16)	27 (84)	0 (0)		
Are you worried about losing your housing?	12 (38)	18 (56)	2 (6)		

© 2025 Long KA et al. *JAMA Network Open*.

eTable 2. Qualitative Interview Questions

Based on the Health Equity Implementation Framework[4,5]

Topic	Lead Interview Questions	Probes
**Experience with HRSN Screening**	To your knowledge, does [name of family medicine clinic] ask about or provide help for meeting needs related to everyday life such as food or housing? If so, please describe the process.	• Tell me about your experience of being asked these questions.<br>• Tell me about your experience of getting information or resources to meet your needs.<br>• What does [name of family medicine clinic] do well when it comes to supporting patients or families to meet their basic needs?<br>• What could [name of clinic] do better?<br>• In your opinion, how important is it for healthcare clinics to help their patients with needs related to everyday life?
**Screening**	I would like to walk through the process of clinics asking about everyday life and needs, and getting support to address those needs.<br><br>The first step usually includes a short form – also called a screener. On this form, patients or families can check off whether they have needs related to everyday life and whether they want support meeting those needs. Here are some examples [show/describe screener examples].<br>**What are your thoughts about these forms?**	• *For each probe, follow up with "how and why" questions to better understand underlying processes*<br>• If you were given this form at [name of clinic], how likely is it that you would fill it out?<br>• How might you decide <u>whether</u> to answer? How might you decide <u>how</u> to answer?<br>• [Innovation]: Thinking about **the form itself** – what might affect <u>whether</u> and <u>how</u> someone might fill it out?<br>• [Recipient – Patient]: What are some things about **patients or families** that might affect <u>whether</u> and <u>how</u> they fill it out?<br>• [Recipient – Providers]: Now thinking about the **clinic staff or healthcare providers** – what things about these professionals might affect <u>whether</u> and <u>how</u> patients or families fill out the form?<br>• [Clinical Encounter]: Thinking about how **patients/families and clinic staff or providers** get along with each other – how might their interaction affect <u>whether</u> and <u>how</u> patients or families fill out the form?<br>• [Context]: Finally, think about **the bigger picture, such as the clinic as a whole, or even things outside of the clinic** – what are some things that might affect <u>whether</u> and <u>how</u> patients or families fill out the form?
**Screener Review & Referral**	After patients or families fill out the form, the next step is that someone from the healthcare office reads the form. Then, they help to connect patients or	• *For each probe, follow up with "how and why" questions to better understand underlying processes*<br>• [Innovation]: Thinking about **this process** in which the clinic staff reads the form and then helps to connect patients/families to services or resources – what might make this process work more or less well?

© 2025 Long KA et al. *JAMA Network Open*.

	families with services or resources. This could be done in a few different ways. For example, someone from the healthcare clinic might provide information about resources to the family or they might connect the patient or family with someone who can help them get services or resources.<br>**What are your thoughts about this part of the process?**	• [Recipient – Patient]: Thinking about the **patients or families** – what things about the patient or family might affect whether and how clinic staff responds to their needs?<br>• [Recipient – Providers]: Now thinking about the **clinic staff or healthcare providers** – what things about these professionals might affect whether they read the form? What are some things about these professionals that might affect whether and how they help patients/families?<br>• [Clinical Encounter]: Thinking about how **patients/families and clinic staff or providers** get along with each other – how might their interaction affect whether clinic staff read the form? How might their interaction affect whether and how the clinic staff or healthcare providers connect patients/families with resources?<br>• [Context]: Finally, think about **the bigger picture, such as the clinic as a whole, or even things outside of the clinic** – what are some things that might affect whether clinic staff read the form? What are some things that might affect whether and how they connect patients/families with resources?
**Connection with Resources**	If patients or families are provided with information about resources or services, the next step is often for the patient or family to reach out to someone in the community.<br>**What are your thoughts about this step in the process?**	• *For each probe, follow up with "how and why" questions to better understand underlying processes*<br>• If you were given a resource sheet like this one [show/describe examples], what might you do with the information?<br>• How might you decide whether to contact the organizations on the resource sheet?<br>• [Innovation]: Thinking about **this process** in which patients/families reach out to the organizations listed on the resource sheet – what might make this process work more or less well? How would you like to get the resource information?<br>• [Recipient – Patient]: Thinking about the **patients or families** – what things about the patient or family might affect whether they reach out to organizations on the sheet?<br>• [Recipient – Providers]: Thinking about the **clinic staff or healthcare providers** – what things about these professionals might affect whether patients/families reach out to organizations on the resource sheets? How about **staff at community organizations**?<br>• [Clinical Encounter]: Thinking about how **patients/families and clinic staff or providers** get along with each other – how might their interaction affect whether patients/families reach out to the organizations on the resource sheet?<br>• [Context]: Finally, think about **the bigger picture, such as the clinic as a whole, or even things outside of the clinic** – what are some things that might affect whether patients/families reach out to organizations on the resource sheet? When they do reach out, what might get in the

© 2025 Long KA et al. *JAMA Network Open*.

		way of patients or families actually getting resources or services? How can more patients receive the support that they want or need?
**Suggestions to Improve**	Taking a step back and thinking about the whole process of asking about challenges of everyday life and obtaining resources or services – **how could this be done better?**	• *For each probe, follow up with "how and why" questions to better understand underlying processes*<br>• **Screening**: Overall, how might we make the screening step better so that more people would fill out the form?<br>• **Review of Screeners:** Overall, how might we make it more likely that clinic staff or providers read the form? … connect patients or families with resources?<br>• **Connecting W/ Resources:** Overall, how might this step be improved so that more patients/families receive the support they want or need?
**Likely Outcomes**	In a perfect world, what would you like to happen as a result of filling out this form?	• *For each probe, follow up with "how and why" questions to better understand underlying processes*<br>• In reality, what is most likely to happen as a result of filling out this form?<br>• [If there are differences between the ideal vs. actual expectations]: What are some reasons that [summarize "perfect world" outcome] might not happen?

© 2025 Long KA et al. *JAMA Network Open*.

eTable 3: Codebook

Code	SubCode	Code Name	Description / Examples
**1**		Step 1: Administering Screener	
	1.1	Previous experience w/ screening	Previous experience **of being asked** about or receiving help for HRSN (i.e., needs related to everyday life such as food or housing), from a screener or screener-like process within a healthcare setting. Note that the emphasis is **on being asked** during screening, rather than the participant getting resources through their own independent effort or advocacy.
	1.1.1	Discussions re: unmet HRSN	How and/or with whom patients discuss their unmet HRSN (emphasis on the **conversation** participants have had related to unmet needs) outside the specific context of screening. Include examples of conversations patients have previously had with doctors, nurses, med assistants, social workers, and/or other healthcare staff. Include descriptions of what made these conversations good/bad. This code can also be used if the participant explicitly articulates the lack of HRSN conversations.
	1.2	General feedback on screening process	General impressions of HRSN screening in primary care and general comments regarding participants' likelihood of engaging with screening. Ex. "I think HRSN screening is a good idea."
	1.3	Screener: Innovation	Comments regarding what might affect whether and/or how someone fills out the screener depending on the screener itself (e.g. content, format). Include responses about the clarity of the screening form & other functional aspects of the screener. Also capture responses of how participants want the form administered to them (e.g., digital vs. paper), including preferences for involved providers/staff (ex. "I would want to fill this out with my doctor").
	1.4	Screener: Recipient-Patient	Aspects of patient or family that might affect whether and/or how patients or families fill out the screener. This includes, but is not limited to, race/ethnicity, presence of personal unmet HRSN, degree of literacy or education, etc.
	1.5	Screener: Recipient-Provider	Aspects of the clinic staff or healthcare providers that might affect whether and/or how patients fill out the screener (e.g., race of provider, role of provider (MD, nurse, etc.)).
	1.6	Screener: Clinical Encounter	How interpersonal interactions between patients/families and clinic staff/providers might affect whether and/or how patients fill out the screener.

© 2025 Long KA et al. *JAMA Network Open*.

	1.7	Screener: Context	Factors within and outside of the clinic that might affect whether and/or how patients fill out the screener. This may include descriptions around how things operate within the clinic (e.g. the culture of the clinic, number of clinic staff members) and outside of the clinic (e.g., participants' eligibility for Medicaid, political considerations city/state/nationwide).
**2**		Step 2: Reviewing Completed Screeners	
	2.1	General feedback on screener review process	General thoughts regarding the **process and idea of reviewing HRSN screeners** and how likely it is that clinic staff/providers read the screeners. Ex "I think they should be reading it"
	2.2	Screener Review: Innovation	Perceptions of how the <u>screener itself</u> may affect how clinic staff review screeners (e.g., length of screener, format of screener, amount of patient information gathered by the screener). Also capture who may best equipped to review HRSN screeners (e.g., social workers, doctors).
	2.3	Screener Review: Recipient-Patient	Aspects of the patient/family that might affect whether and/or how clinic staff/providers review screeners (e.g., reported HRSN, race/ethnicity).
	2.4	Screener Review: Recipient-Provider	Aspects of the clinic staff/providers that might affect whether and/or how clinic staff/providers review screeners (e.g., role of provider (MD, nurse, etc.), staff's personal experience of HRSN).
	2.5	Screener Review: Clinical Encounter	How interactions between patients/families and clinic staff/providers might affect whether and/or how staff/providers review screeners (e.g., how previous positive or negative interactions affect urgency and depth of screener review)
	2.6	Screener Review: Context	Factors within and outside of the clinic that might affect whether and/or how clinic staff/providers review screeners. This may include descriptions around how things operate within the clinic (e.g. the culture of the clinic, number or responsibilities of clinic staff members) and outside of the clinic (e.g., clinic funding, political considerations city/state/nationwide).
**3**		Step 3: Receiving Resource Sheets	
	3.1	General feedback on receiving resource sheets	General thoughts regarding the **process and idea of distributing HRSN-related resource sheets**. Include descriptions of what participants might do with the information on the resource sheet and how they might decide whether or not to contact the organizations on the resource sheet.
	3.2	Resources: Innovation	Descriptions regarding the <u>resource sheet itself</u> and what about it may hinder/improve the process of patients/families reaching out and/or connecting to resources via organizations listed on the resource sheet. Examples may include the types of resources included on resource sheets, the format and content of the information presented on the sheet (e.g., length, language), and any preferences on

© 2025 Long KA et al. *JAMA Network Open*.

			how to receive information about community-based resources or organizations (e.g., via email vs. hard copy, by certain staff members).
	3.3	Resources: Recipient-Patient	Aspects of the patient/family that might affect whether and/or how they reach out to—or are otherwise connected with—HRSN-related resources (e.g. languages spoken, literacy, immigration status).
	3.4	Resources: Recipient-Clinic Provider	Aspects of the clinic staff/providers that may affect whether and/or how patients/families reach out to—or are otherwise connected with—HRSN-related resources (e.g., race of provider, role of provider).
	3.4.1	Resources: Recipient-Other Providers	Aspects of staff outside of clinics (e.g., staff at community-based resource organizations) that may affect whether and/or how patients/families reach out to—or are otherwise connected with—HRSN-related resources (e.g. staff member's race, ethnicity, language knowledge, cultural competency).
	3.5	Resources: Clinical Encounter	How interactions between patients/families and clinic staff/providers might affect whether and/or how patients/families reach out to—or are otherwise connected with—HRSN-related resources (e.g., provider recommendations to connect with 'X' resource). This may include thoughts on whether/how participants would like a clinic staff member to follow-up to see if they were able to connect with an organization and receive resources.
	3.6	Resources: Context	Factors within and outside of the clinic that might affect whether and/or how patients/families reach out to—or are otherwise connected with—HRSN-related resources. This may include descriptions around how things operate within the clinic (e.g. the culture of the clinic, number of clinic staff members) and outside of the clinic (e.g., available resources in a given community, political considerations city/state/nationwide).
**4**		General Recommendations	
	4.1	Recommendations for addressing unmet needs	Any other, non-screening specific, thoughts/ideas for how to improve the process of asking patients about their HRSN and/or facilitating the process of providing resources or services that patients or families want or need (e.g., hosting a clinic resource event, in-clinic food pantry)
	4.2	Expectations for outcomes of the whole HRSN screening & referral process	Description of ideal and actual expectations following completion of the screener, as well as clinic/staff involvement. Also include perceived benefits or losses from making personal HRSN known and/or asking for support in primary care settings.

© 2025 Long KA et al. *JAMA Network Open*.

eReferences

1. Hager ER, Quigg AM, Black MM, et al. Development and validity of a 2-item screen to identify families at risk for food insecurity. *Pediatrics*. 2010;126(1):e26–e32.
2. Frank DA, Casey PH, Black MM, et al. Cumulative hardship and wellness of low-income, young children: Multisite surveillance study. *Pediatrics*. 2010;125(5):e1115–e1123.
3. Sandel M, Sheward R, Ettinger de Cuba S, et al. Unstable housing and caregiver and child health in renter families. *Pediatrics*. 2018;141(2):e20172199.
4. Woodward EN, Singh RS, Ndebele-Ngwenya P, Melgar Castillo A, Dickson KS, Kirchner JE. A more practical guide to incorporating health equity domains in implementation determinant frameworks. *Implement Sci Commun*. 2021;2(1):61. Published 2021 Jun 5. doi:10.1186/s43058-021-00146-5
5. Woodward EN, Matthieu MM, Uchendu US, Rogal S, Kirchner JE. The health equity implementation framework: Proposal and preliminary study of hepatitis C virus treatment. *Implement Sci*. 2019;14(1):26. Published 2019 Mar 12. doi:10.1186/s13012-019-0861-y

© 2025 Long KA et al. *JAMA Network Open*.
