# Supplementary material for: Perspectives on Health-Related Social Needs Screening in Primary Care Among Black and Latine Patients
Source: JAMA Netw Open. 2025 Aug 28;8(8):e2529538. doi: 10.1001/jamanetworkopen.2025.29538 (PMC12395316; doi:10.1001/jamanetworkopen.2025.29538)
Supplement: Supplement 2. — Data Sharing Statement [file jamanetwopen-e2529538-s002.pdf]

Data Sharing Statement

Long. Perspectives on Health-Related Social Needs Screening in Primary Care Among Black and Latine Patients. *JAMA Netw Open*. Published August 28, 2025.
doi:10.1001/jamanetworkopen.2025.29538

Data
Data available: No

Additional Information
Explanation for why data not available: Due to the combination of small sample sizes in qualitative research, the possibility that participant identity may become apparent from narratives even when transcripts are carefully deidentified, and the sensitive nature of the personal data disclosed, we will not be making qualitative datasets public.
